# Base editing-coupled survival screening enabled high-sensitive analysis of PAM compatibility and finding of the new possible off-target

**DOI:** 10.1016/j.isci.2021.102769

**Published:** 2021-06-24

**Authors:** Tianyuan Su, Qi Guo, Yi Zheng, Yizhao Chang, Fei Gu, Xuemei Lu, Qingsheng Qi

**Affiliations:** 1 State Key Laboratory of Microbial Technology, Shandong university, Qingdao 266237, People's Republic of China; 2 CAS Key Lab of Biobased Materials, Qingdao Institute of Bioenergy and Bioprocess Technology, Chinese Academy of Sciences, Qingdao 266101, People's Republic of China

**Keywords:** Molecular biology, Systems biology

## Abstract

Base editing (BE) is a promising genome engineering tool for modifying DNA or RNA and has been widely used in various microorganisms as well as eukaryotic cells. Despite the proximal protospacer adjacent motif (PAM) is critical to the targeting range and off-target effect of BE, there is still lack of a specific approach to analyze the PAM pattern in BE systems. Here, we developed a base editing-coupled survival screening method. Using dCas9 from *Streptococcus pyogenes* (SpdCas9) and its variants xdCas9 3.7 and dCas9 NG as example, their PAM patterns in BE systems were extensively characterized using the NNNN PAM library with high sensitivity. In addition to the typical PAM recognition features, we observed more unique PAMs exhibiting BE activity. These PAM patterns will boost the finding of potential off-target editing event arising from non-canonical PAMs and provide the guidelines for PAM usage in the BE system.

## Highlights

• BESS method was proposed to focus and characterize the functional PAMs in BE
• New non-canonical PAMs were identified to have BE activity
• PAM patterns identified by BESS warn of the potential off-target editing

## Graphical abstract

**Figure:**
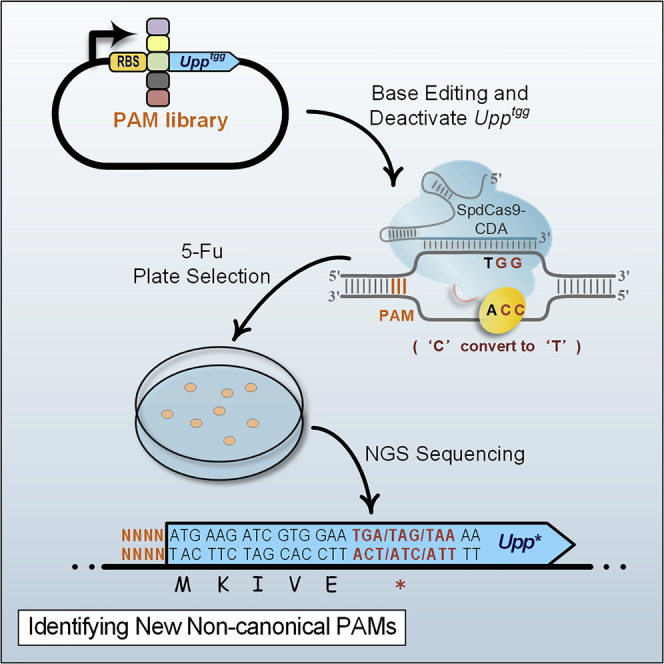

## Introduction

Base editing (BE) is an important genome editing technique that harnesses catalytically impaired dead Cas9 (dCas9) or nicking Cas9 (nCas9)-guided nucleotide modification enzymes to modify of specific bases (Komor et al., 2016; Nishida et al., 2016). In contrast to Cas9 endonuclease-mediated homologous recombination (Zhou et al., 2019b), the BE system based on dCas9 does not cause DNA double-strand breaks (DSBs), which are highly genotoxic for the host, and is therefore regarded as a more promising genome editing tool for applications in basic research and gene therapy (Mention et al., 2019; Rees and Liu, 2018). However, in addition to interrogating DNA sequences that are complementary to sgRNA, the complexes must recognize a short protospacer adjacent motif (PAM) located upstream of the DNA target sequence for efficient BE (Jinek et al., 2012; Mojica et al., 2005). PAM compatibility not only determines the sequence accessibility in the BE system, the recognition of non-canonical PAMs also would increase the risk of potential off-target editing.

The most commonly applied Cas9 nuclease from *Streptococcus pyogenes* (SpCas9) recognized NGG as the optimal PAM (canonical PAM) (Mali et al., 2013). Several non-canonical PAMs including NAG, NGA, and NCG have also been reported to support cleavage of DNA by SpCas9, albeit less efficiently than the canonical NGG PAM (Hu et al., 2019; Jiang et al., 2013). The extensive PAM recognition will not only expand the genomic target scope but also may increase the risk of off-target editing. Tsai et al. have reported that non-canonical PAMs such as NAG, NGA, NAA, NGT, NGC, and NCG would trigger genome-wide off-target DSBs (Tsai et al., 2015). However, researchers generally used the canonical NGG PAM in the SpdCas9-based BE system. It is currently unknown whether the BE system can use other non-canonical PAMs and result in off-target editing. On the other hand, off-target editing is the major bottleneck for the reliable application of BE, especially for gene therapy (Fu et al., 2013; Lin et al., 2014; Liu et al., 2018). Therefore, it is very essential to comprehensively characterize the usefulness of various PAMs and evaluate the off-target risk in the BE system.

Several methods have been developed to determine the PAM sequences for Cas9 nuclease or its variants (Leenay and Beisel, 2017). These methods can be divided into four categories: (1) in silico identification of PAMs with the natural CRISPR array sequences through Basic Local Alignment Search Tool (BLAST) search and flanking sequence alignment (Savitskaya et al., 2013). (2) *In vitro* DNA cleavage assay using the purified Cas proteins, *in vitro* transcribed guide RNAs, and a target DNA library with randomized PAM sequences (Karvelis et al., 2015). PAMs that depleted after the cleavage reaction are considered to be functional PAMs. (3) *In vivo* DNA cleavage assay, also known as plasmid clearance assay (Jiang et al., 2013). The Cas effector proteins, guide RNAs, and the target plasmid library with randomized PAM sequences were expressed in a convenient host and subjected to the cleavage reaction *in vivo*. The plasmids with functional PAMs would be cleared and show a significant decline in the high-throughput sequencing results (Esvelt et al., 2013). (4) PAM SCreen Achieved by NOT-gated Repression (PAM-SCANR) and PAM-SEARCH are a group of recently developed methods that *in vivo* analyze the DNA binding ability of dCas with various PAM sequences (Collias et al., 2020; Leenay et al., 2016). By far, the most common used method is the *in vivo* plasmid clearance assay, which recapitulated the natural feature of CRISPR-Cas system to clean of foreign genetic elements (Elmore et al., 2015; Esvelt et al., 2013; Jiang et al., 2013). However, BE only requires the DNA binding of dCas9, not the DNA cleavage activity. Therefore, the cleavage-based PAM determination method is not well suited for the BE system. PAM-SCANR enables rapid elucidation of the functional PAMs supporting the DNA binding of dCas9 that based on the NOT-gate repression of fluorescent protein through fluorescence-activated cell sorting (Leenay et al., 2016). Some new functional PAMs for *B. halodurans* type I-C CRISPR system have been identified using this method. In addition to DNA binding by dCas9, BE relies on the base modification enzyme, such as the cytosine deaminase or adenine deaminases (Gaudelli et al., 2017; Komor et al., 2016), to modify the specific bases. None of these methods can directly reveal the gene editing results of BE, as well the potential risk of off-target editing, for different PAM sequences.

Here, we developed a base editing-coupled survival screening method (BESS) and analyzed the PAM compatibility of SpdCas9 and its PAM-broadened variants in the BE system. Most importantly, because of enrichment and screening of the base-edited mutants with 5-fluorouracil (5-Fu), BESS is able to identify certain PAMs with less BE activity in high sensitivity, which is extremely helpful for studying the off-target effect of BE.

## Results

### Design of the base editing-coupled survival screening

**Figure 1:**
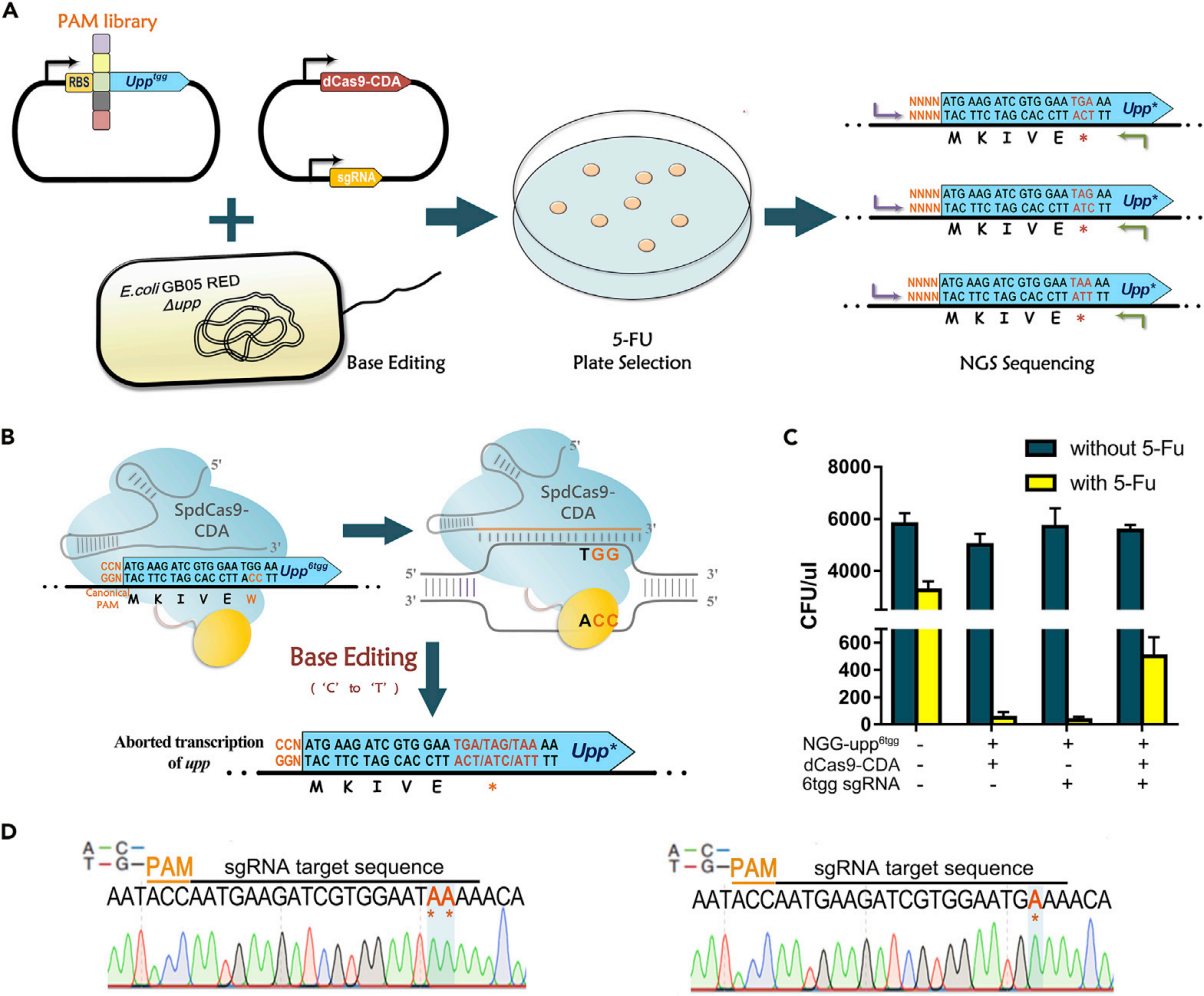
Base editing-coupled survival screening (BESS) (A) Schematic for BESS. An NNNN random PAM library upstream of *upp*^*6tgg*^ gene was transferred into GB05Red Δ*upp* with the cytimidine base editor (CBE). Functional PAMs allow C⋅G to T⋅A editing of *upp*^*6tgg*^ and terminate its expression. The *upp*-inactivated strain will be enriched by 5-Fu screening, and the PAM patterns can be analyzed according to NGS sequencing results. (B) Principle of deactivating *upp* by CBE. In the presence of a functional PAM sequence (such as the canonical NGG PAM), CBE can convert the TGG codon in *upp*^*6tgg*^ to TAG, TGA or TAA, which all encoded termination codons, and inactivate the expression of *upp*. (C) Editing of NGG-*upp*^*6tgg*^ in GB05Red Δ*upp* strain and enable its growth on 5-Fu plates. Error bars represent the standard deviation (SD) of three independent biological replicates. (D) Sanger sequencing of *upp*^*6tgg*^ variant demonstrated C⋅G to T⋅A base conversation in the TGG codon.

To evaluate the high-sensitive PAM pattern, we designed a cytimidine BESS (simplified as “BESS”) (Figure 1A). In this method, the counter selective gene *upp* was modified into *upp*^*6tgg*^ by replacing the sixth amino acid valine (encoded by GTC) with a tryptophan (encoded by TGG) (Figure S1). 5-Fu plate experiment demonstrated that the *upp*^*6tgg*^ variant has the same 5-Fu sensitivity as the original *upp* gene (Figure S2). If the inserted TGG codon in *upp*^*6tgg*^ mutated into TAA, TGA, or TAG that all encode termination codons by the cytimidine base editor (CBE) (Banno et al., 2018), the translation of *upp*^*6tgg*^ would be prematurely terminated and the strain will grow on 5-Fu screening plates (Figure 1B); otherwise, 5-Fu is lethal to the strain containing *upp*^*6tgg*^ (Andersen et al., 1992).

To prove this concept, the CBE system consisting of the cytosine deaminase PmCDA1 fused with SpdCas9 (SpdCas9-CDA) was introduced into *Escherichia coli* strain GB05Red Δ*upp*. In the absence of sgRNA, strain GB05Red Δ*upp* with NGG-*upp*^*6tgg*^ could only grow on LB plates without 5-Fu (Figure S2), and after importing sgRNA and inducing CBE, approximately 10% of the cells were able to grow on the 5-Fu plates (Figures 1C and S3). Sanger sequencing showed that 11 of 16 random selected colonies grown on 5-Fu plates contained C⋅G-to-T⋅A base conversation at the third position of TGG codon, and the remaining strains had CC in the antisense chain of TGG mutated to TT (Figure 1D). These demonstrated that BESS can edit NGG-*upp*^*6tgg*^ and confer strain 5-Fu resistance.

### Enrichment capability of BESS for functional PAMs

**Figure 2:**
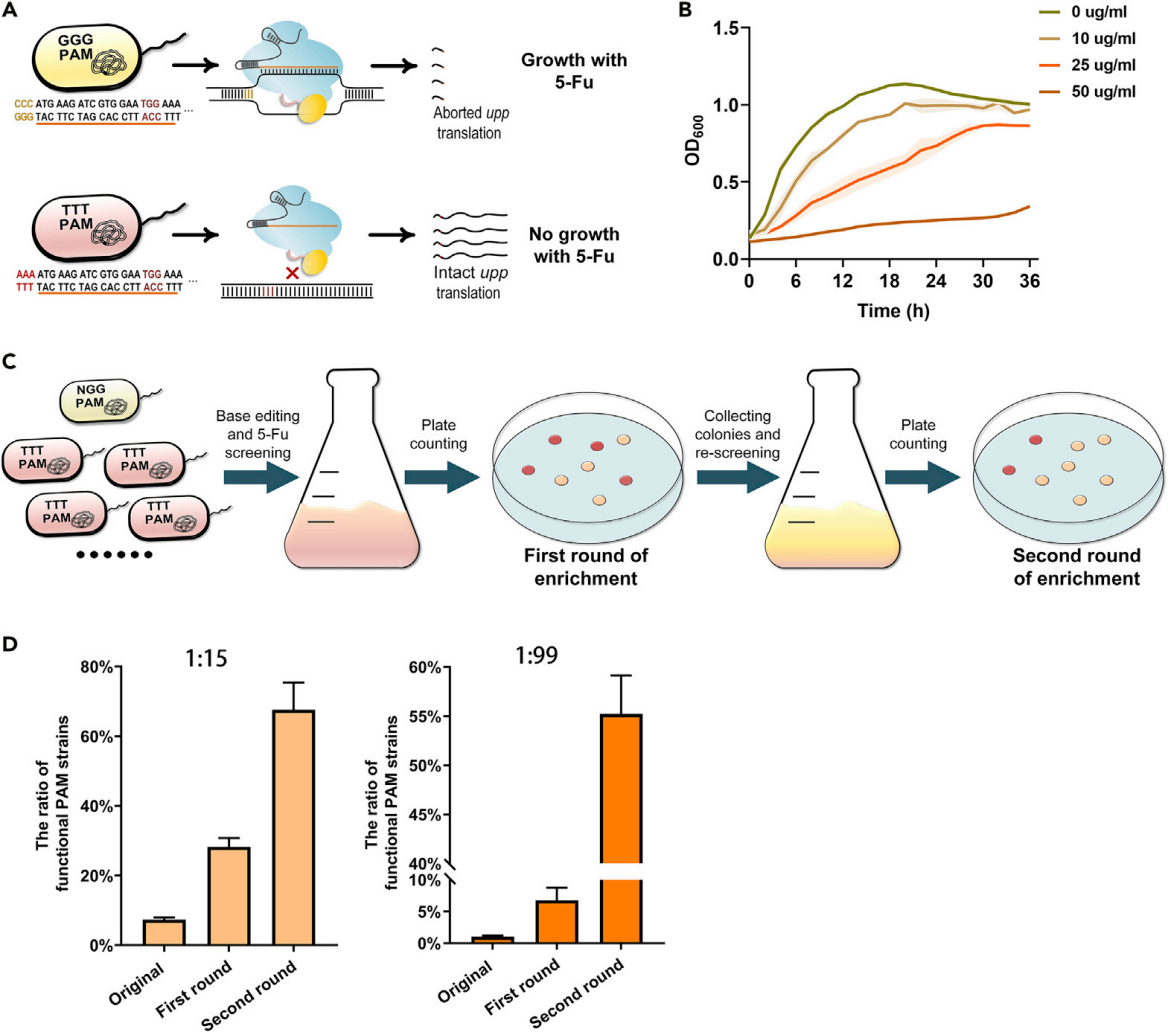
The performance of BESS to ensrich functional PAM sequences (A) Illustration of *upp*^6tgg^ expression with the functional PAM (GGG) and invalid PAM (TTT) after BE. (B) Growth curves of the *upp*-deficient strain GB05Red Δ*upp* with various 5-Fu concentrations. The shaded areas represent the standard deviation (SDs) of three independent biological replicates. (C) Illustration of screening and enriching functional PAMs by BESS. The enrichment of functional PAMs by BESS can be visualized by the color of colonies growing on the 5-Fu plates. (D) Simulating enrichment of functional PAMs by BESS. To evaluation the enrichment capability of BESS for the PAM library, the functional NGG PAM strain was purposely mixed with the negative TTT PAM strain at the original ratio of 1:15 or 1:99 for BESS screening. The ratio of functional PAM strain was determined by the percentage of red colonies on the 5-Fu plates. Error bars represent the standard deviation (SD) of three independent biological replicates.

The major advantage of BESS is the efficient enrichment of base-edited mutants through 5-Fu screening. Functional PAMs, such as NGG, are able to edit *upp*^6tgg^ and terminate its translation. However, invalid PAMs, such as TTT, cannot mutate *upp*^6tgg^ (Figure 2A). The growth of Δ*upp* strain was slightly impaired in 10 μg/mL of 5-Fu, while the wild-type strain was already unable to grow at the same 5-Fu concentration (Figures 2B and S4). This indicates that only strains carrying functional PAMs and causing nonsense mutation in *upp*^6tgg^ can be enriched by 5-Fu screening.

To evaluate the enrichment capability of BESS for the functional PAM sequences, strain GB05Red Δ*upp* harboring CBE and *upp*^*6tgg*^ with the canonical NGG PAM was mixed with the same strain but containing an invalid TTT PAM sequence at a ratio of 1:15. Besides, to visually distinguish the NGG PAM strain, the TTT PAM strain was co-expressed with a red fluorescent protein (Mkate) (Figure 2C). This model can reveal the screening effectiveness of BESS if only NGG in the NNN random PAM library, 1/16 of the total, is able to inactivate *upp*^*6tgg*^. When the mixture was induced, the percentage of NGG PAM strain identified by colony counting increased from 7.3% to 28.1% after 24 hr of cultivation in liquid medium with 10 μg/ul of 5-Fu. After harvesting the colonies for another round of enrichment, the proportion of NGG PAM strain increased to 67.6%, 9-fold enrichment compared to the starting mixture (Figure 2D).

To further investigate the sensitivity of BESS, the ratio of functional PAM strain to invalid PAM strain was set to 1:99. With CBE and 5-Fu screening, the proportion of functional PAM strain increased from 1.0% to 6.8%, and after a second round of screening, the proportion even increased to 55.2%, 55 folds enriched than the initial proportion (Figure 2D). This proved that BESS can be used to efficiently enrich strains with functional PAM sequences in the BE system.

### BESS revealed extensive, non-canonical PAMs for SpdCas9-CDA

The above results showed that BESS can be used to enrich and evaluate the activity of functional PAMs. As the most prevalent Cas effector protein used in the BE system (Rees and Liu, 2018; Zhao et al., 2020), the PAM compatibility of SpdCas9 was first subjected to BESS screening. To do that, NNNN PAM libraries containing 256 various PAM sequences were constructed ahead the target site of *upp*^*6tgg*^, forming 6TGG PAM library. Four nucleotides were introduced between the Shine Dalgarno region and the coding sequence, which possibly impacts the translation of *upp*^6tgg^. We co-expressed a β-lactamase in tandem with *upp*^6tgg^ during the construction of the PAM library and balanced the impact of the inserted sequence on translation by imposing ampicillin pressure. The NGS sequencing results showed that the inserted NNNN sequences did not cause significant negative consequences on gene expression, and the PAM sequences were normally distributed in the initial PAM library after ampicillin selection (Figure S5).

**Figure 3:**
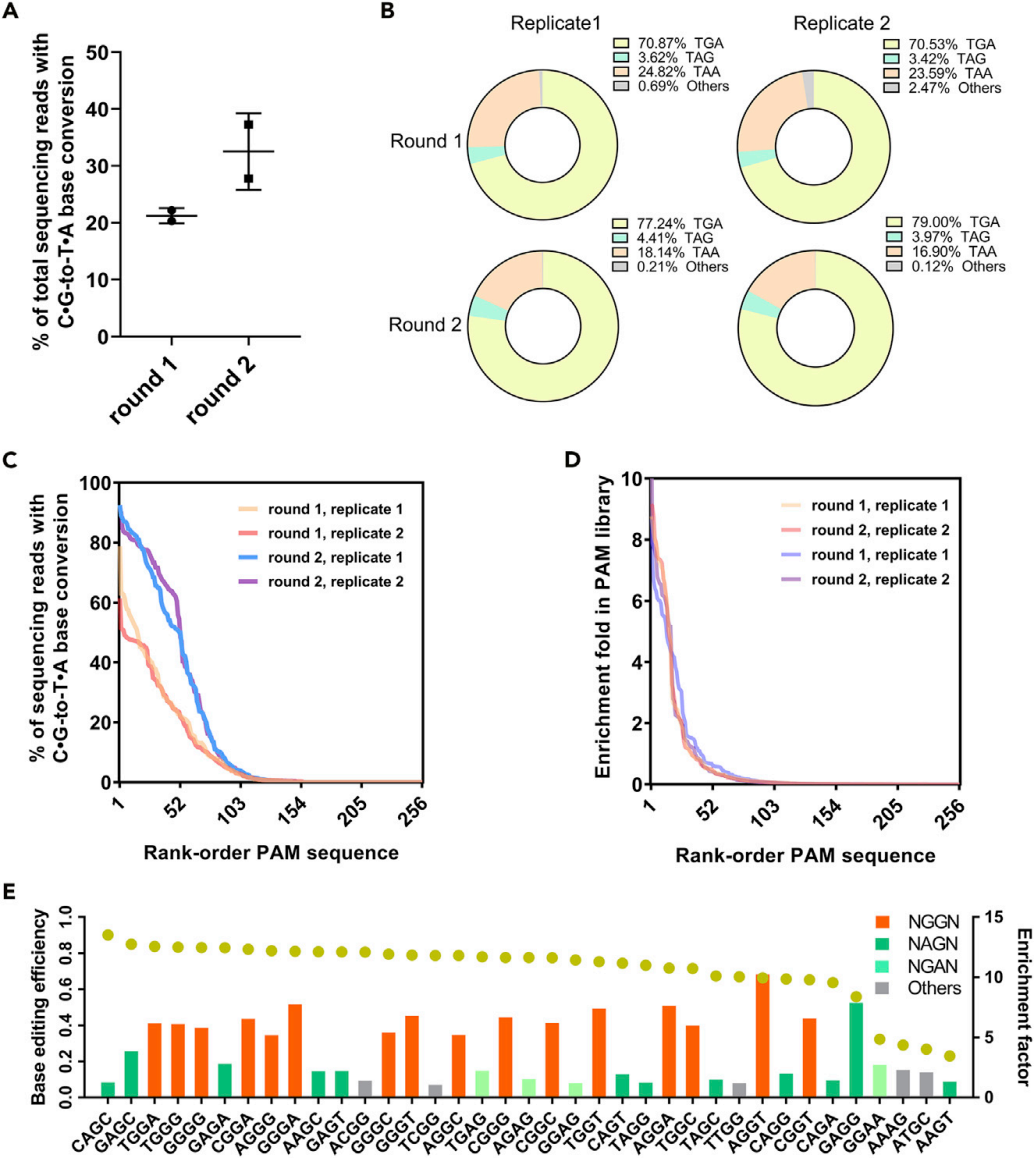
Enrichment of functional PAMs for SpdCas9-CDA by BESS (A) Overall C⋅G to T⋅A base conversion ratios of the NNNN PAM library in the two rounds of BESS. Error bars represent the standard deviation (SD) of two independent biological replicates, with individual values shown as dots. (B) The mutation patterns of TGG codon in *upp*^6tgg^. (C) The overall C⋅G to T⋅A base conversion ratios for PAMs in the two rounds of BESS screening with two biological replicates. (D) The overall PAM enrichment profiles in the two rounds of BESS screening with two biological replicates. (E) Functional PAMs with enrichment factor greater than 1 after two rounds of BESS screening. Brown points represent BE efficiencies and the histograms indicate enrichment factors.

Then, 6TGG PAM library was introduced into the GB05Red Δ*upp* strain for BESS. After two rounds of screening, the 152 bp DNA fragment containing the PAM and sgRNA targeting sequence was PCR amplified for high-throughput sequencing (Figure 1A). For the first round of enrichment, an average of 19.8% sequencing reads appeared C⋅G-to-T⋅A base conversion at the TGG codon. After second round of enrichment, the proportion increased to 37.2% (Figure 3A). Among these mutant reads, TGG mutate to TGA accounted for the highest proportion, followed by TAA, and TAG was the least (Figure 3B). Besides, there were no significant changes in the mutation patterns as the rounds of enrichment increased. These results are consistent with the previously reported one that cytosine at the 18^th^ of sgRNA is more efficient relative to the 17^th^ in the CBE system (Nishida et al., 2016).

In the two biological replicates, there were 122 and 118 PAMs detected with more than 1% of sequencing reads carrying the C⋅G-to-T⋅A base conversion on the TGG codon (Figure 3C). That means almost half of PAMs in the NNNN PAM library can undergo BE. This amount is far beyond the original consensus that SpCas9 only recognizes a few PAM sequences such as NGG, NAG, and NGA. Although the number of functional PAMs did not increase with the number of BESS rounds, the overall percentage of the base-edited reads for these functional PAMs significantly increased after the second round of BESS enrichment (Figure 3C). These results demonstrated that BESS can effectively enrich functional PAMs with BE activity among the PAM library, and as the rounds of BESS screening increased, the BE efficiency of these PAMs would be further improved.

### One base shift of the core PAM sequence supports SpdCas9 for BE

After two rounds of BESS, 37 and 42 PAMs were enriched in the two duplicate PAM libraries, respectively (Figure 3D). These enriched PAM sequences included all of the 16 NGGN sequences, 14 NAGN sequences, 4 NGAN, and 6 other sequences (Figures 3E and S6, Data S1). Among them, all NGG sequences had the enrichment factors greater than 5 and BE efficiencies higher than 77%, indicating that NGG was the most effective PAM sequence for SpdCas9 in the BE system (Figures 3E and S6). In addition, we found that four of the six other PAM sequences can be categorized into NNGG, and the BE efficiency of ACGG, TCGG, and TTGG even exceeded 70% (Figure 3E). To further confirm this result, *upp*^6tgg^ with the ACGG, TTGG, or GTGG PAM sequence was separately constructed for BE experiment. After 24 hr of induction, nonsense mutations of *upp*^6tgg^ were observed in these strains, demonstrating that certain NNGG sequences enabled to support SpdCas9 for BE (Figure S7).

**Figure 4:**
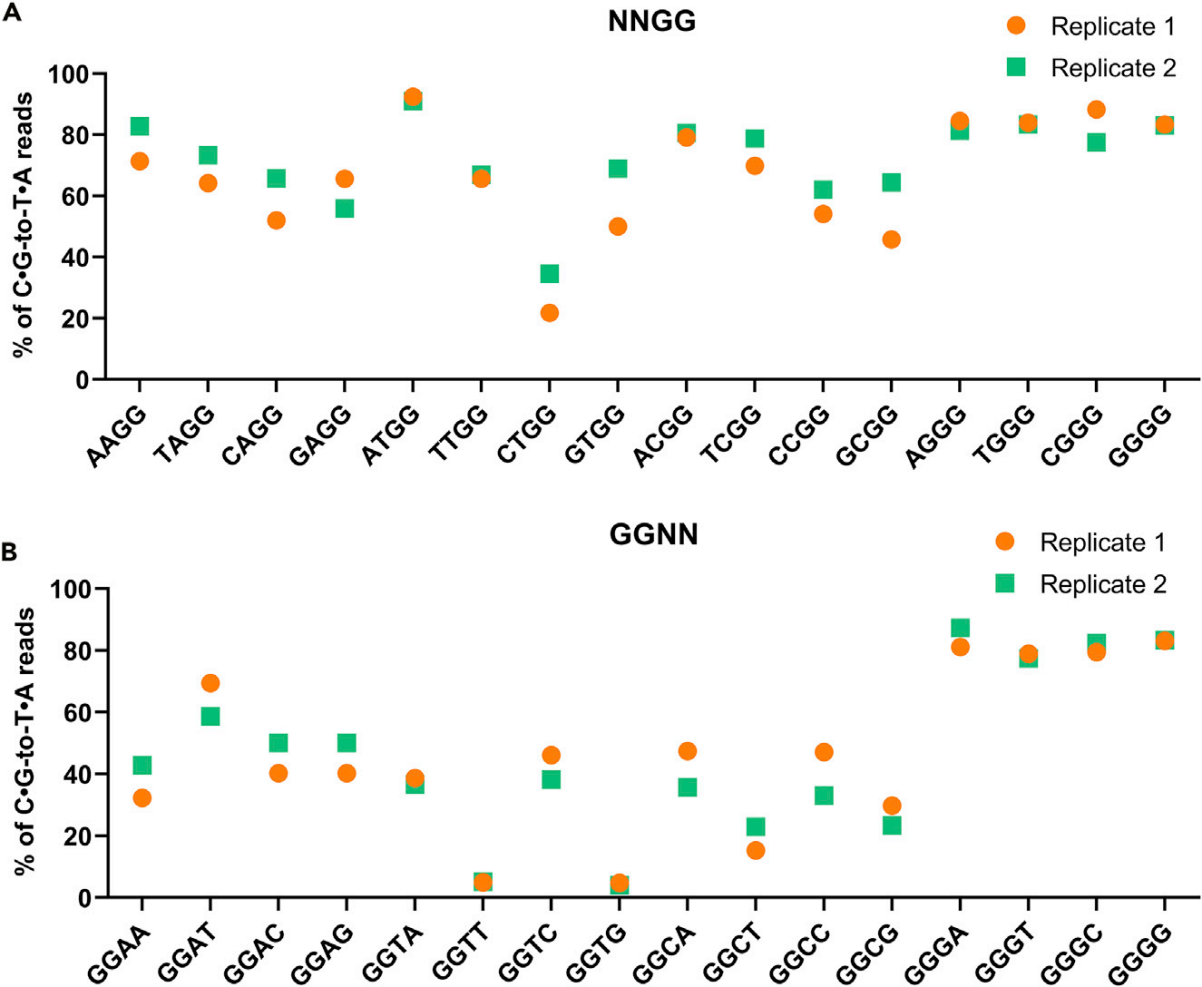
NNGG and GGNN sequences could serve as functional PAMs for BE The ratios of C⋅G to T⋅A base conversion for *upp*^6tgg^ with NNGG (A) and GGNN (B) PAM sequences after two rounds of BESS screening in the two biological replicates.

It has been reported that SpCas9 was able to utilize NNGG PAM to cleave a certain target DNA with limited activity in bacteria and eukaryotic cells and had the potential off-target editing risk (Jiang et al., 2013; Kim et al., 2020). BESS screening revealed that all NNGG sequences could serve as functional PAMs for BE (Figure 4A). In addition to the well-known functional NAGG and NGGG PAMs, all NTGG and NCGG have more than 20% of sequencing reads that showed C⋅G-to-T⋅A base conversion. In particular, the BE efficiency of ATGG even reached 91%, which is higher than that of the canonical NGG PAM. This result implied that SpdCas9 appeared to be able to tolerate one base backward shift of the core NGG PAM. Since BE relies on the DNA binding capacity of dCas9, we investigated the performance of NNGG as the PAM sequences in CRISPRi assay that also only require DNA binding of dCas9. CRISPRi experiment also demonstrated that most of the NNGG sequences, except GCGG and CAGG, were able to repress the expression of *gfp* in varying degrees (Figure S8).

Importantly, we found that one base forward of the NGGN PAM, namely GGNN, was capable of converting cytosine to thymine at the TGG codon as well (Figure 4B). Although GGCN and GGTN were less efficient than GGAN and GGGN, but with the exception of GGTT and GGTG, all of the remaining GGNN PAMs showed C⋅G-to T⋅A-base conversation in more than 20% of the sequencing reads. These demonstrated that SpdCas9 can actually tolerate one base forward or backward shift of the NGG PAM in the BE system.

### Identification of more unnoticed PAMs with potential BE activity

**Figure 5:**
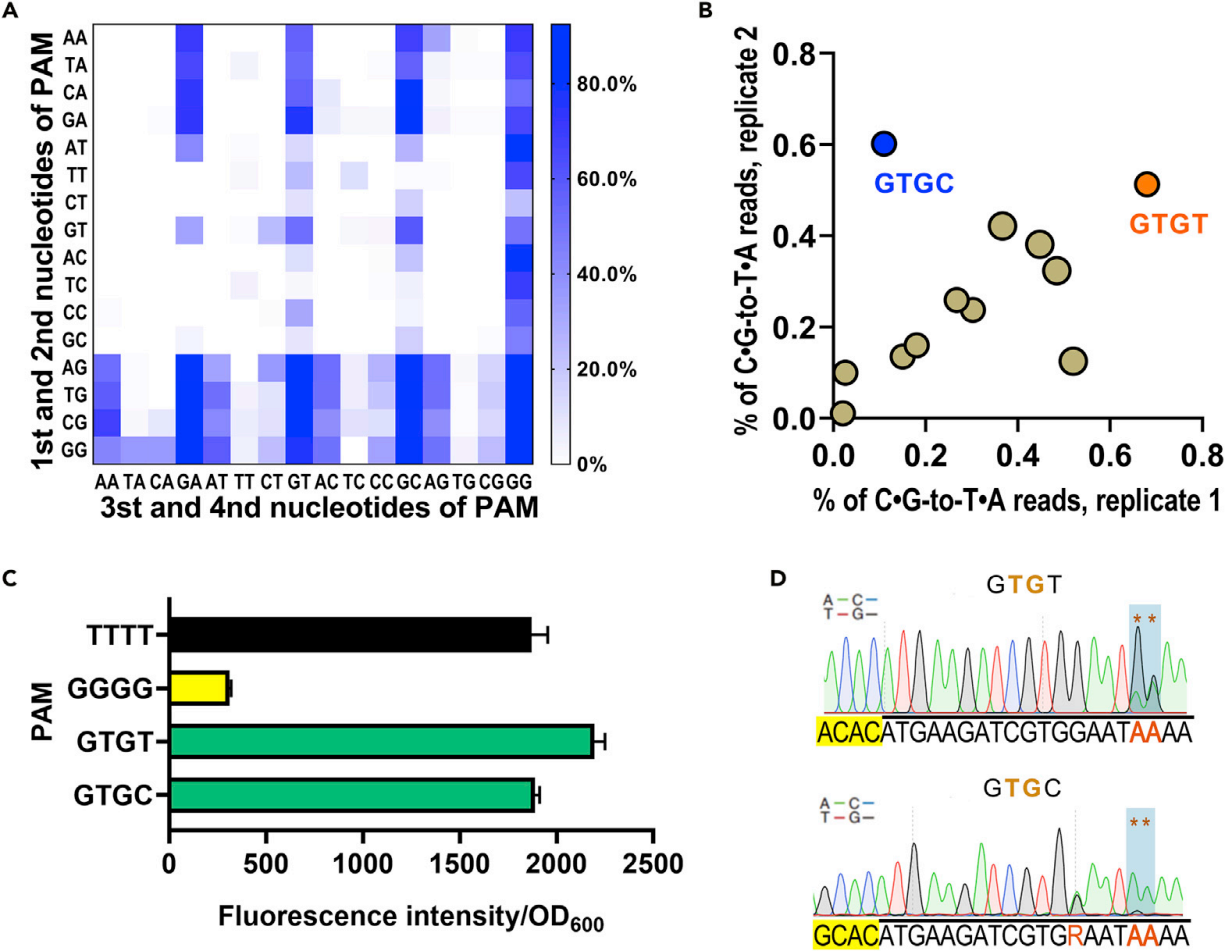
Potential PAMs for SpdCas9 in the BE system identified by BESS (A) Heatmap evaluation of BE efficiency for the 256 different PAM sequences. The editing frequencies were normalized to the highest frequency in each heatmap. Full data can be found in Data S1. (B) The BE activity of the NTGN sequence in the two biological replicates. GTGC and GTGT sequence with BE efficiencies over 60% were marked in blue and orange, respectively. (C) GTGT and GTGC as PAM sequences to repress the expression of GFP by CRISPRi. TTTT and GGGG sequences were used as negative and positive controls, respectively. Error bars represent the standard deviation (SD) of three independent biological replicates. (D) Sanger sequencing of the target TGG codon in *upp*^6tgg^ after induction of BE using the potential GTGT or GTGC PAMs.

The above BESS results showed that in addition to NGG, SpdCas9 can utilize a number of other PAMs, such as NAG, NGA, NNGG, and GGNN, for efficient BE. However, these 65 PAMs only account for about half of the BESS-identified PAMs that with BE activity (Figure 3C). There are still a large number of previously unnoticed potential PAM sequences that have been identified by BESS screening (Data S1). In order to comprehensively investigate the PAM pattern of SpdCas9, we plotted the BE efficiency heatmap for the 256 different PAM sequences (Figure 5A). Sequence logos indicated that the best PAM for SpdCas9 identified by BESS was NGG (Figure S9), which was consistent with the previous reported one (Jiang et al., 2013; Mojica et al., 2009). We found that the PAM sequences of NGCB (B = G/T/C), NTGN, NCGY (Y = T/C), GTCT, TTTC, and AAAG also allowed SpdCas9 to edit of *upp*^6tgg^ at different degrees (Figure 5A). The editing efficiency of certain NTGN PAMs was even comparable to that of the canonical NGG PAM, with a maximum of about 60% (Figure 5B). It is worth to note that these PAM sequences have never been reported to support SpdCas9 for BE.

The PAM recognition properties of SpCas9 have been extensively studied with various methods (Karvelis et al., 2015; Leenay et al., 2016). We thus intended to investigate whether a similar pattern was evidenced in the previously published data sets. The most widely used plasmid clearance experiment showed significant depletion of NGG in the library, and certain NAG and NNGG sequences also had some degree of depletion (Jiang et al., 2013) (Figure S10). However, other potential PAMs identified by BESS could not be captured in this DNA cleavage-based negative selection method. Recently, the BE activity of SpdCas9 with various PAM sequences has been extensively characterized in 46 sgRNA target sites (Miller et al., 2020). Due to the lack of selection and enrichment for the BE events, only 4.4% of sequencing reads on average yielded C⋅G-to-T⋅A base conversation at the target sites, much lower than the 37.2% of overall BE frequency in the second round of BESS (Figure 3A). Comprehensive evaluation of the PAM pattern of these 46 sgRNA target sites revealed that NGCG and GTGN had weak BE activity, which is consistent with our results identified by BESS (Figures 5A and S10). However, most previously unnoticed PAMs were not captured in this PAM library but identified by BESS, possibly due to the weak sensitivity to the low-activity PAMs.

To validate the PAM pattern obtained by BESS, two potential PAMs, GTGC and GTGT, with the highest base conversion efficiency among NTGN sequence (Figure 5B), were individually placed in front of *upp*^6tgg^ and used to demonstrate the BE activity. After 12 hr of incubation, colonies emerged on the 5-Fu plates. Sanger sequencing demonstrated that both of GTGC and GTGT had actually supported SpdCas9 to generate C⋅G-to-T⋅A base conversion on the TGG codon of *upp*^6tgg^ (Figure 5D). This is the first report that certain NTGN sequences can serve as PAMs for SpdCas9 to base edit the target DNA. Notably, CRISPRi experiments showed that neither GTGT nor GTGC could support SpdCas9 for efficiently repressing the transcription of target gene (Figure 5C). The results suggested that certain potential PAMs might only activate SpdCas9-CDA to transiently, rather than persistently, bind the target DNA and convert C⋅G to T⋅A in some particular conformations. This is why other PAM determination methods fail to identify these potential PAMs that only support BE.

To further demonstrate the effectiveness of the identified PAMs, sgRNA sequences with different PAMs were designed to introduce the termination codon into the genomic *lacZ* gene through the C⋅G-to-T⋅A base conversion by BE. We constructed a total of nine sgRNA sites for the three major groups of PAMs identified by BESS (Figure S11), including the NGCB PAMs (TGCT, CGCC, TGCC), NTGN PAMs (ATGT, GTGC, CTGC), and NCGY PAMs (GCGC, CCGC, CCGT). All the sgRNA sites were able to efficiently inactivate *lacZ* in the *E. coli* MG1655 strain as shown by blue-white screening (Figure S12) and Sanger sequencing proved that the expected cytosines in the *lacZ*-inactivated strains were converted to thymines (Figure S13).

### Comprehensive evaluation of the PAM patterns for the Cas9 variants with BESS

Recently, more and more Cas9 variants with broader PAM recognition capabilities have been developed and used in BE (Hu et al., 2018; Nishimasu et al., 2018; Walton et al., 2020). To further confirm the effectiveness of BESS, PAM patterns of the two more representative Cas9 variants, xCas9 3.7 and Cas9 NG (Hu et al., 2018; Nishimasu et al., 2018), were comprehensively characterized with the NNNN PAM library. After replacing dCas9 with its variants, PAM sequences capable of editing *upp*^6tgg^ were enriched by BESS screening. NGS sequencing showed that 29.0% and 35.3% of sequencing reads for xdCas9 3.7-CDA and dCas9 NG-CDA showed C⋅G-to-T⋅A base conversation on the target TGG codon (Data S1).

**Figure 6:**
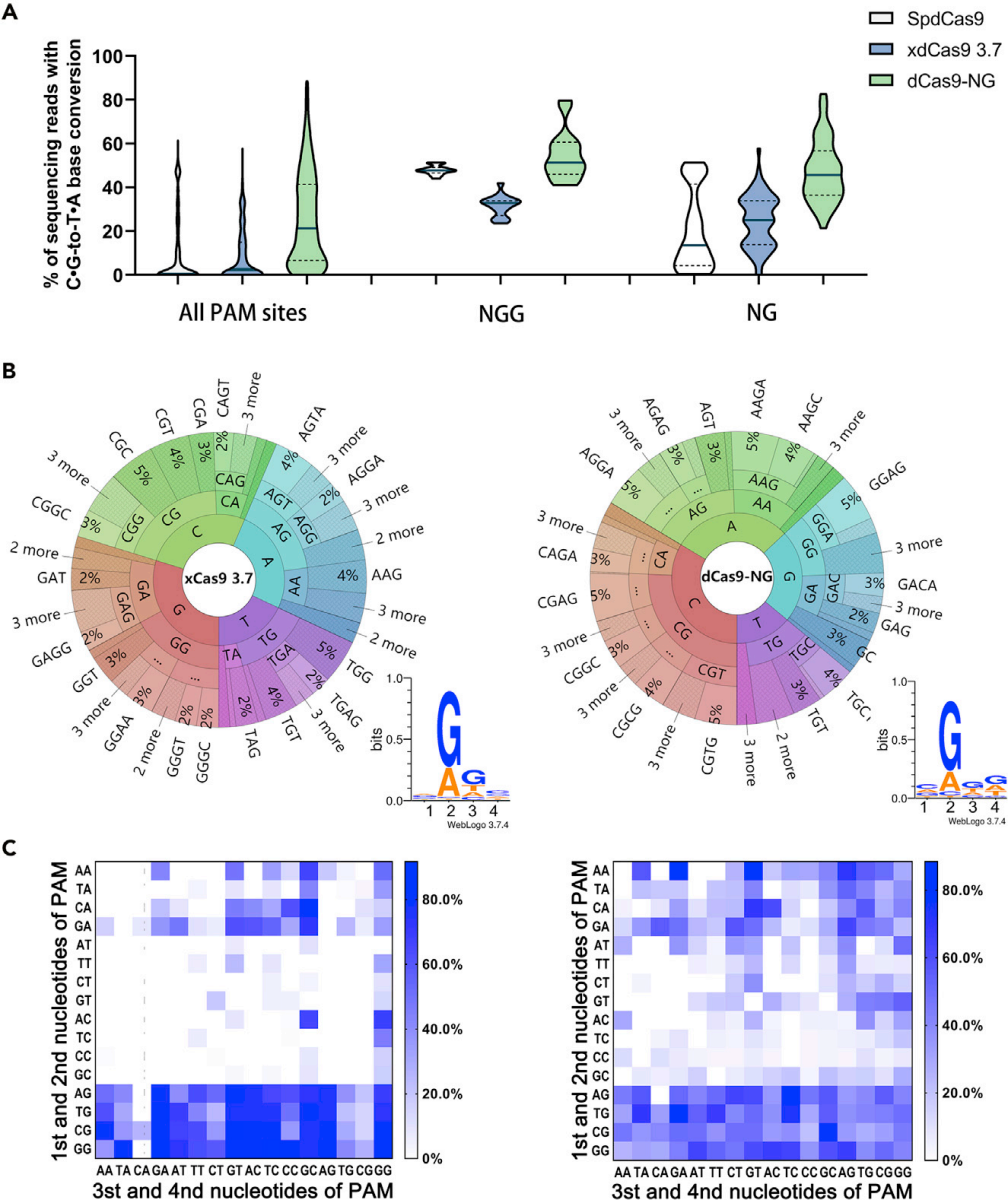
PAM patterns identified by BESS for xdCas9 3.7-CDA and dCas9 NG-CDA (A) Summary of the overall C⋅G to T⋅A base conversation ratios for SpdCas9, xdCas9 3.7, and dCas9 NG base editors on NNNN, NGG, or NG PAM sequences. For each construct, the BE efficiency across all the designated PAMs in the library is shown, with solid lines indicating median and dotted lines indicating first and third quartiles. (B) Visualization of the base composition for functional PAMs via PAM Wheel and sequence logos. (C) Heatmap evaluation of the BE efficiencies for xdCas9 3.7-CDA (left) and dCas9 NG-CDA (right). The editing frequencies were normalized to the highest frequency in each heatmap. The exact values can be found in Data S1.

BESS screening results indicated that the overall base conversion ratio of dCas9 NG-CDA in the NNNN PAM library was obviously higher than that of SpdCas9-CDA and xdCas9 3.7-CDA (Figure 6A). For the NGG PAMs, dCas9 NG-CDA was also the most efficient, followed by SpdCas9-CDA, and xdCas9 3.7-CDA was the lowest. It has been reported that both dCas9 NG and xdCas9 3.7 can effectively recognize NG as PAM sequence. We found that dCas9 NG can efficiently utilize all of the NG sequences to edit the target DNA. However, despite recognizing more NG sequences than SpdCas9, xdCas9 3.7 was still inefficient for part of these PAM sequences (Figure 6A).

The detailed base composition of functional PAMs was visualized by sequence logo and PAM wheel (Figure 6B). Sequence logos indicated that the second and third bases of the PAM sequence for xdCas9 3.7 were more preferred to guanine, whereas unlike the original SpdCas9, guanine preference for the third base is slightly less than that for the second base (Figures 6B and S9). For dCas9 NG, only the second base has a clear preference for guanine and adenine, while other positions did not exhibit obvious base preference (Figure 6B). It should be noticed that the sequence logos did not completely represent the details of the PAM patterns. PAM wheel is a more powerful PAM representation scheme that is able to convey both individual sequences and the corresponding enrichment parameter in the PAM library (Leenay et al., 2016). With PAM wheel, we found that although the first position of PAM sequence usually had no base preference, it can influence the composition of the subsequent bases. For instance, if the first base is thymine, the second base is more likely to be guanine, regardless of xdCas9 or dCas9 NG (Figure 6B). This means that TANN is less efficient than the other VAHH (V = G/A/C) PAMs, that is, the specificity of TG PAMs is better than that of the other VG PAMs in the BE system.

The heatmap revealed that the NG sequences for xdCas9 3.7-CDA, with the exception of DGCA (D = A/T/G), had varying degrees of BE activity. Among them, the efficiency of NGG, NGA, and NGTC was significantly higher than that of other PAMs (Figure 6C). PAM depletion assay showed that xCas9 3.7 is able to cleave the target DNA with NG, NNG, GAA, GAT, and CAA PAMs (Hu et al., 2018). However, our assay showed that xdCas9 3.7 is unable to use all of the GAT, CAA, and NG PAMs for BE. Only CAAT and CAAC among CAAN, NG excluding DGCA, as well as GATN excluding GATA, were able to undergo BE with the efficiency varying from 9.24% to 91.71% (Data S1). Interestingly, we found that most NANC sequences exhibited notable BE activity as well (Figure 6C). For the other variant, dCas9 NG, a more broad-ranging recognition of PAMs was identified by BESS. We found that in addition to the well-known NG PAMs (Nishimasu et al., 2018), dCas9 NG was also able to recognize most of the NA sequences. Furthermore, only 48 of the 256 random PAM sequences were completely unable to support dCas9 NG for BE, while 161 of the remaining functional PAMs exhibited BE efficiencies greater than 10% (Data S1). In particular, more than half of the NTNT, NTNG, and NCNG sequences showed significant C⋅G-to-T⋅A base conversion. It appeared that only the NTNA region generally could not allow dCas9 NG for BE. Although it is difficult to further generalize a deeper pattern of PAM recognition for dCas9 NG, the BESS results at least demonstrated that dCas9 NG can recognize more PAMs in the BE system than previously expected, which allows it to access and edit more DNA sequences.

Together, these data sets showed that BESS has the capability to efficient couple BE with *upp*-based counter screening, which can be used for analysis of the complex PAM patterns for the SpdCas9 variants with high accuracy and sensitivity.

## Discussion

Recognition of PAM is a prerequisite for CRISPR-Cas system to target specific DNA sequences. Despite BE has been broadly used for genome engineering for more than four years (Komor et al., 2016; Nishida et al., 2016), the PAM pattern for SpdCas9 has not been completely clarified in the BE system. Recently, an increasing number of SpCas9 variants with varied PAM preferences or target specificity have also been developed and applied to BE (Miller et al., 2020; Nishimasu et al., 2018; Walton et al., 2020). However, the PAM compatibility of these variants was typically determined by DSB-based plasmid clearance assay and not fully suitable for the BE system which only relied on the DNA binding of dCas9. It is highly desirable to establish a more specialized method that allows us to comprehensively characterize the PAM patterns of these variants in the BE system. A cytosine base editor (CBE) high-throughput PAM determination assay was reported to rapidly profile the PAM preferences of SpCas9 variants in the BE system (Walton et al., 2020). In this method, the BE reaction was performed *in vitro* with conditions approximating a human cell context. David R. Liu et al. characterized the BE results *in vivo* on 38538 genomically integrated targets and proposed a machine learning model (BE-Hive) to predict BE efficiency and editing patterns (Arbab et al., 2020). While these results faithfully reveal the BE efficiency of different PAM sequences, it is likely to be ignored for a few non-canonical PAM sequences because of the poor BE activity. In this work, we proposed a BE-coupled survival screening method. Because of the BE activity-dependent survival screening prior to high-throughput sequencing analysis, BESS is more conducive to enrich and analyze low BE activity PAMs with high sensitivity.

Similar high-throughput approaches have recently been reported to analyze the indels generated by SpCas9 or its variants with various PAMs (Kim et al., 2020; Tang et al., 2019). With the exception of NGG, NAG, NGA, NNGG, and GGCN PAMs that were identified to be able to generate indel in human cells, we for the first time observed certain previously unnoticed PAM sequences, such as NGCB (B = G/T/C) and NYGY (Y = T/C), that support SpdCas9 for efficient BE. The difference in the PAM preference results may be due to two reasons. First, the nuclease-dependent indel mutations by Cas9 may have more strict PAM requirements than the BE system, which only requires the DNA binding capability of dCas9 (Rees and Liu, 2018). It has been reported that using the nicking version of Cas9 to excise the unedited DNA strand could improve the BE efficiency but as well increasing the risk of unexpected indels (Lei et al., 2018). In contrast, the BE system used in this work only relies on dCas9 to bind of DNA and pull of the fused deaminase to the target DNA region, which does not cleave DNA and generate DSBs. Therefore, the unique PAM pattern identified by BESS may only allow SpdCas9 to bind DNA rather than efficiently cleavage of DNA and generate indel mutations. Another reason is that the conventional analysis methods may be prone to miss certain poor activity PAMs due to the lack of screening for the mutants. For the study of off-target effects, these omissions are very detrimental. It has been reported that the addition of a functional reporter system, such as the fluorescent protein, would be more beneficial in screening for cells with BE at the target site (Katti et al., 2020; St Martin et al., 2018; Standage-Beier et al., 2020). In BESS, a similar BE activity-dependent survival screening was implemented prior to high-throughput sequencing, which was able to enrich functional PAMs with low BE activity. Without such screening, those low-activity PAMs are probably ignored in the sequencing results when the most of them are not edited. Although the additional screening might aggravate the spontaneous mutations of *upp*, however, we did not find any other abnormal mutations in the high-throughput sequencing results except for the programmed C-to-T base substitutions.

Most of the knowledge on how SpCas9 nuclease recognizes of PAM sequence has been determined so far (Anders et al., 2014). Two conserved arginine residues, Arg1333 and Arg1335 in SpCas9, can interact with the GG dinucleotide and position the target DNA duplex (Figure S14). The +1 phosphate group of the target strand subsequently interacts with the phosphate lock loop and facilitates DNA strand unwinding (Anders et al., 2014). It has been reported that one base shift of the core PAM sequence, namely GGNN or NNGG, can also allow SpCas9 cleavage of the target DNA (Chen et al., 2014; Collias et al., 2020; Kim et al., 2020; Zeng et al., 2018). This is consistent with our finding that NNGG and GGNN can support SpdCas9 for BE. However, there are still certain new potential PAMs identified by BESS that cannot be explained by PAM shifting. We found that a common feature among these functional NGCB (B = G/T/C) and NYGY (Y = T/C) PAMs was that at least one guanine appeared at the second or third position. Moreover, it has been demonstrated that in addition to NGG, PAM sequences such as NAG and NGA are also capable of directing SpCas9 to cleave target DNA with reduced efficiency (Jiang et al., 2013). Therefore, we speculate that as long as the presence of one guanine at either the second or third position of PAMs interacts with Arg1333 or Arg1335, it may confer the possibility for SpdCas9 to bind to the target DNA sequence and allow the base modification enzyme to modify the corresponding bases in certain particular conformation (Figure S14). Besides, a small amount of non-NGG PAM for SpCas9 was also founded when in silico analysis of PAM sequence using the natural CRISPR array data (Biswas et al., 2013), providing additional evidence to support this hypothesis. However, the crystal structure of SpCas9 with the HNH domain in an active conformation has not been observed so far (Zhang et al., 2020); more works are still needed to clearly resolve the PAM recognition and functional mechanism for the Cas9 nuclease.

Predicting and avoiding off-target editing is of great importance for the application of BE in precise gene therapy (Bolukbasi et al., 2016; Doman et al., 2020). The off-target effects in BE can be classified into two types according to the dependence of sgRNA (Yang and Chen, 2020). The sgRNA-independent off-target effect is caused by the intrinsic property of cytidine deaminase, which can randomly bind and deaminate the intracellular RNA or ssDNA substrates (Jin et al., 2019; Zuo et al., 2019). Such genome-scale off-target editing would be unpredictable and might be alleviated by engineering of the cytidine deaminases (Zhou et al., 2019a). In contrast, sgRNA-dependent off-target effect is mainly attributed to mismatches of sgRNA with the genomic non-targeted DNA sequences, which are, to some extent, predictable and can be avoided by rational design of sgRNA (Hwang et al., 2021). In this study, non-canonical PAMs identified by BESS have been validated to efficiently cause BE in different loci of the *E. coli* genome. The loose PAM requirements identified by BESS probably increase the possibility for SpdCsa9 to unravel DNA duplexes and generate “R-loop” in more genomic regions. Therefore, we suggest that in addition to the sgRNA mismatches at the canonical PAM, the non-canonical PAMs with potential BE activity also require attention when assessing the off-target effect of BE.

### Limitations of the study

Here we developed a simple and sensitive method to analyze the BE activity for various PAM sequences in *E. coli*. Although it has not been reported that the PAM patterns of BE system varied among species, the PAM recognition properties obtained in this work remain to be validated when adapted to eukaryotic cells.

## STAR★Methods

### Key resources table

**Table:** 

REAGENT or RESOURCE	SOURCE	IDENTIFIER
**Bacterial and virus strains**		

*E. coli* DH5α	Thermofisher	Cat#: EC0112
*E. coli* MG1655	ATCC	ATCC700926
*E. coli* BW25141	CGSC	CGSC#:7635
GB05Red	(Wang et al., 2014)	N/A
REDd	This paper	N/A
REDx	This paper	N/A
REDng	This paper	N/A

**Deposited data**		

Raw deep-sequencing data	This paper	NCBI BioProject: PRJNA657496

**Recombinant DNA**		

pCP20	(Datsenko and Wanner, 2000)	N/A
pTKRED	(Datsenko and Wanner, 2000)	N/A
pKD4	(Datsenko and Wanner, 2000)	N/A
pHK	(Haldimann and Wanner, 2001)	N/A
pAH69	(Haldimann and Wanner, 2001)	N/A
pgRNA-bacteria	(Qi et al., 2013)	N/A
p15A-gRNA	(Su et al., 2016)	N/A
pSC-upp^6tgg^	This study	N/A
Rd	This study	N/A
Rx	This study	N/A
Rng	This study	N/A
PAM library	This study	N/A
pSC-NGG-upp^6tgg^	This study	N/A
pTTT-UPP^6TGG^-MKATE	This study	N/A
pBE	This study	N/A
TGCT-sgRNA	This study	N/A
CGCC-sgRNA	This study	N/A
TGCC-sgRNA	This study	N/A
ATGT-sgRNA	This study	N/A
GTGC-sgRNA	This study	N/A
CTGC-sgRNA	This study	N/A
GCGC-sgRNA	This study	N/A
CCGC-sgRNA	This study	N/A
CCGT-sgRNA	This study	N/A
6tgg-sgRNA	This study	N/A
pUPP^6TGG^-AmpR	This study	N/A
upp^6tgg^-NNNN	This study	N/A
pdCas9	This study	N/A
pSC-TTTT-GFP	This study	N/A
pSC-NNGG-GFP	This study	N/A
pSC-GTGT-GFP	This study	N/A
pSC-GTGC-GFP	This study	N/A

**Software and algorithms**		

Weblogo 3.0	(Crooks et al., 2004)	http://weblogo.threeplusone.com/
Sequence analysis script	This study	Data S2
PAM wheel	(Leenay et al., 2016)	N/A
PyMOL	Schrödinger	https://pymol.org/2/

### Resource availability

#### Lead contact

Further information and requests for resources and reagents should be directed to and will be fulfilled by the lead contact, Qingsheng Qi (qiqingsheng@sdu.edu.cn).

#### Materials availability

This study did not generate new unique reagents.

#### Data and code availability

All data supporting the results are available within the paper and its supplemental information files. The raw deep-sequencing data are available at the NCBI BioProject under the accession number PRJNA657496.

### Experimental model and subject details

#### Microbial strains

The *upp* gene in *E. coli* GB05Red (Wang et al., 2014) was knockout by the one-step gene inactivation method described previously (Datsenko and Wanner, 2000). The BE element Ptet-SpdCas9-CDA, Ptet-xdCas9 3.7-CDA or Ptet-Cas9 NG-CDA was separately integrated into the genome of GB05Red Δ*upp* using the phage integrase (Haldimann and Wanner, 2001). The resulting strains REDd, REDx and REDng were used for the BE experiments. 1μM anhydrotetracycline (aTc) was added at an OD_600_ of approximately 0.4 to induce base editing. 50 mg/L of 5-Fu was used for screening of *upp* mutant. E. coli DH5α was used as the host for cloning experiments. All of the strains were grown in Luria-Bertani (LB) medium (10 g/L tryptone, 5 g/L yeast extract, and 5 g/L NaCl) supplemented with appropriate antibiotic (ampicillin (100 mg/L), kanamycin (25 mg/L), chloramphenicol (25 mg/L), and spectinomycin (50 mg/L)).

### Method details

#### Plasmid construction

The counterselective gene *upp* was PCR (2×T5 Super PCR Mix, TSINGKE) amplified from the genome of E. coli MG1655 and adapted to *upp*^*6tgg*^. The *upp* variant was then cloned into the low-copy pSC101 vector with the P23119 promoter and NGG PAM by Gibson cloning. The negative control plasmid pTTT-UPP^6TGG^-MKATE was modified from pNGG-UPP^6TGG^ by replacing the PAM sequence from NGG to TTT, and expressing an additional red fluorescent protein (Mkate). The PAM library template plasmid pUPP^6TGG^-AmpR was derived from pNGG-UPP^6TGG^ by co-expressing with the ampicillin resistance gene AmpR using p23119 promoter. The BE elements SpdCas9-CDA, xdCas9 3.7-CDA and dCas9 NG-CDA were commercially synthesized by GENERAL BIOL and cloned into the oriRγ plasmid pHK with Ptet promoter. The sgRNA sequence targeting *upp*^6tgg^ was inserted into p15A-gRNA via golden gate assembly (Su et al., 2016). The sgRNA sequence targeting genomic *lacZ* gene was inserted into pgRNA-bacteria (Qi et al., 2013) by Gibson cloning. To validate the repression of GFP using various PAM sequences, SpdCas9 was cloned into pBAD24, generating pdCas9. A total of 49 6tgg-GFP expression plasmids containing TTTT, NNGG, GTGT or GTGC PAMs were constructed by Gibson cloning. All the plasmid constructed were confirmed via sanger sequencing. DNA sequences of the core elements used in this study were listed in Table S1.

#### PAM library construction

To construct the initial NNNN PAM plasmid library, pUPP^6TGG^-AmpR was used as the DNA template for reverse PCR (Phanta Super-Fidelity DNA Polymerase, vazyme) and the NNNN sequence was added at the appropriate position before *upp*^6tgg^. The resulting amplicons containing DNA homologous sequences were purified using 1% agarose gel and self-assembled by Gibson cloning at 50°C for 1 hr. The products were then transformed into the electrocompetent cells DH5α by MicroPulser (Bio-Rad). After 1 hr incubation, the transformed cells were spread on the LB agar plates containing ampicillin and incubated at 37°C for 12 hr. Expression of ampicillin resistance gene AmpR and *upp* variants within the same operon can ensure the integrity of promoter and RBS for *upp* variants in the library under ampicillin screening. At least 10^5^ clones were collected from the plates and mixed in liquid medium for 12 hr cultivation. The library plasmids were purified from the cultures using a Plasmid Maxiprep kit (TIANprep Mini Plasmid Kit, TIANGEN).

#### Base editing-coupled survival screening

For validation of BESS, pSC-NGG-upp^6tgg^ and the sgRNA expressing plasmid 6tgg-sgRNA were co-transformed into REDd (GB05Red Δ*upp* with genomic integration of SpdCas9-CDA). The resulting strain was added 1μM aTc at OD_600_ of 0.4 for BE of *upp*^6tgg^. After 6hr incubation, 10 ul of inoculum was spread on the screening plate containing 50 μg/mL of 5-Fu and incubated at 37°C for 20 hr. C⋅G to T⋅A mutation in *upp*^6tgg^ was confirmed by sanger sequencing.

To illustrate the screening ability of BESS, REDd containing 6tgg sgRNA and pSC-NGG-upp^6tgg^ was mixed with the same strain that containing 6tgg sgRNA and pTTT-UPP^6TGG^-MKATE at the rate of 1:15 or 1:99. BESS was conducted with the mixed strains as described above. The screening efficacy was calculated based on the proportion of red strains on 5-Fu plates.

To characterize the PAM compatibility of SpdCas9, xdCas9 3.7 or dCas9 NG in BE system, 2μg of NNNN PAM library plasmids were transformed into the electrocompetent cells REDd, REDx or REDng with their sgRNA by MicroPulser. 1μM of aTc was added into the resulting strains for base editing. After 24hr incubation with 10 μg/mL 5-Fu, 1 mL of inoculum was spread on the 25 cm 5-Fu solid plate and incubated at 37°C for 20 hr. Six clones were randomly selected for sanger sequencing to assess the generation of C-T mutations. Then, at least 10^5^ clones on the plate were harvested for another round of BESS. All clones grown on 5-Fu plates were collected into the liquid medium for 2 hr cultivation. The enriched PAM library plasmids were purified from the cultures using a Plasmid Maxiprep kit.

#### Deep sequencing and data analysis

For determination of the PAM library coverage, the 152 bp DNA sequence containing NNNN PAM (as seen in Table S1) was PCR amplified using the initial PAM library plasmids as template in 50 μL reaction volumes. The resulting PCR products were gel purified using a DNA purification kit (TIANgel Midi Purification Kit, TIANGEN). A total of 100 ng purified amplicon DNA was prepared for high-throughput sequencing using the NovaSeq platform (GENEWIZ). At least 1 GB of sequencing data was acquired for each sample. Raw reads were cleaned by removing adapter sequences, ploy-N and low-quality sequences. The clean data of fastq format were used to analyze the number and frequency of different PAM sequences in the library by the Perl script (Data S2).

For analysis of the enriched functional PAM sequence, the 152 bp DNA sequence containing NNNN PAM (as seen in Table S1) was PCR amplified using the enriched PAM library plasmids as template in 50 μL reaction volumes. A total of 100 ng purified amplicon DNA was prepared for high-throughput sequencing using the NovaSeq platform (GENEWIZ). At least 3 GB of sequencing data was acquired for each sample. The C⋅G to T⋅A mutation with various functional PAM sequences in the library were analyzed using the clean sequencing data by the Perl script (Data S2). The enrichment factor is defined as the proportion of a certain PAM sequence that occurring BE in the enriched PAM library to the proportion of the corresponding PAM in the original library. Sequence logos for the functional PAM sequences were produced by the on the online website http://weblogo.threeplusone.com/ (Crooks et al., 2004). The PAM wheel was generated using the enrichment factor for each functional PAM (Leenay et al., 2016).

#### Validation of the identified PAMs

To test the BE effectiveness of the identified PAMs in the *E. coli* genome, sgRNA expression plasmid TGCT-sgRNA, CGCC-sgRNA, TGCC-sgRNA, ATGT-sgRNA, GTGC-sgRNA, CTGC-sgRNA, GCGC-sgRNA, CCGC-sgRNA, and CCGT-sgRNA were respectively transformed into the *E. coli* MG1655 strain containing the pBE plasmid to editing of the genomic *lacZ* gene. The resulting strain was added 0.2% arabinose at OD_600_ of 0.4 to induce the expression of BE system. After 6hr incubation, 10 ul of inoculum was spread on the blue-white screening plate and incubated at 37°C for 20 hr. The C⋅G to T⋅A base conversion in *lacZ* gene was further confirmed by sanger sequencing.

### Quantification and statistical analysis

All of the statistical details can be found in the figure legend and method details section of STAR methods.

## Supplemental information

Document S1. Figures S1–S14

Table S1. DNA sequences of the core elements in this study, related to the section of plasmid construction in the STAR method s

Data S1. Summary of the NGS sequencing data, related to Figures 3, 5, and 6

Data S2. The Perl script for Data analysis, related to Figures 3, 5, and 6

## Footnotes

Supplemental information can be found online at https://doi.org/10.1016/j.isci.2021.102769.
