# Supplementary material for: Base editing-coupled survival screening enabled high-sensitive analysis of PAM compatibility and finding of the new possible off-target
Source: iScience. 2021 Jun 24;24(7):102769. doi: 10.1016/j.isci.2021.102769 (PMC8324807; doi:10.1016/j.isci.2021.102769)
Supplement: Document S1. Figures S1–S14 [file mmc1.pdf]

iScience, Volume *24*

Supplemental information

Base editing-coupled survival screening enabled high-sensitive analysis of PAM compatibility and finding of the new possible off-target

Tianyuan Su, Qi Guo, Yi Zheng, Yizhao Chang, Fei Gu, Xuemei Lu, and Qingsheng Qi

Content

Figure S1. Illustration of the *upp*^{6tgg} sequence.

Figure S2. The 5-Fu sensitivity of GB05, GB05 Δ*upp*, and GB05 Δ*upp* expressing the *upp*^{6tgg} variant.

Figure S3. Base editing of the *upp*^{6tgg} variant.

Figure S4. Growth curves of the strain GB05Red in various 5-Fu concentrations.

Figure S5. The diversity and abundance of PAM sequence in the initial PAM libraries.

Figure S6. Functional PAM sequences with enrichment factor greater than 1 after two round of BESS screening (replicate 2).

Figure S7. Sanger sequencing of *upp*^{6tgg} after BE using the ACGG, GTGG, and TTGG PAMs.

Figure S8. NNGG as PAM sequences to repress the expression of GFP by CRISPRi.

Figure S9. Sequence logos for the functional PAM sequences of SpdCas9 identified after two round of BESS screening (two replicates).

Figure S10. Heat map evaluation of the PAM compatibility with plasmid clearance experiment (A) and BE activity experiment (B).

Figure S11. The distribution of sgRNA sequences with various PAMs on the lacZ gene.

Figure S12. Blue-white screening of strains carrying sgRNA targeting lacZ gene with different PAMs after BE.

Figure S13. Sanger sequencing of the target sites on the genomic *lacZ* gene after BE using sgRNA with different PAMs.

Figure S14. Interaction of arginine at the 1333 and 1335 positions in SpCas9 with NGG PAM.

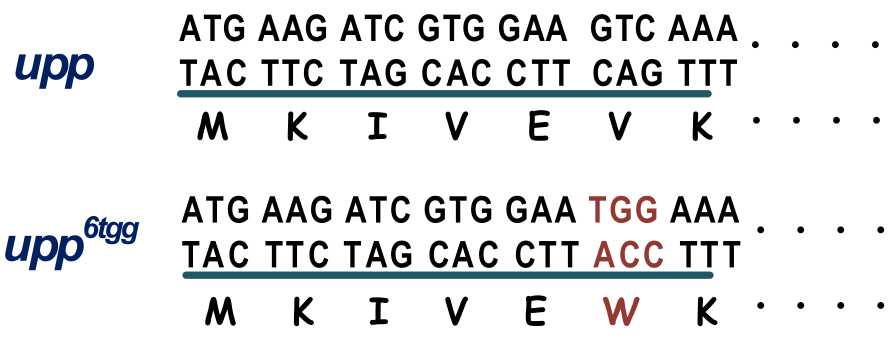

Figure S1. Illustration of the *upp*^{6tgg} sequence. The engineered amino acid is marked in red and the sgRNA targeting sequences are underlined in blue (Related to Figure 1).

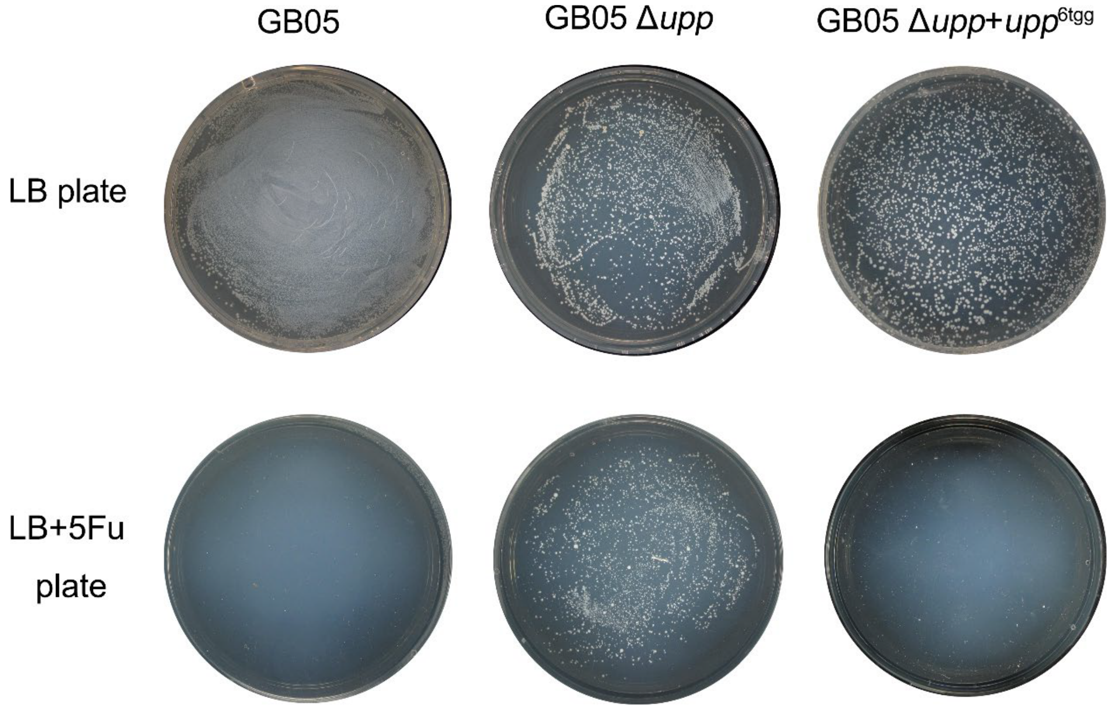

Figure S2. The 5-Fu sensitivity of GB05, GB05 Δ*upp*, and GB05 Δ*upp* expressing the *upp*^{6tgg} variant (Related to Figure 1).

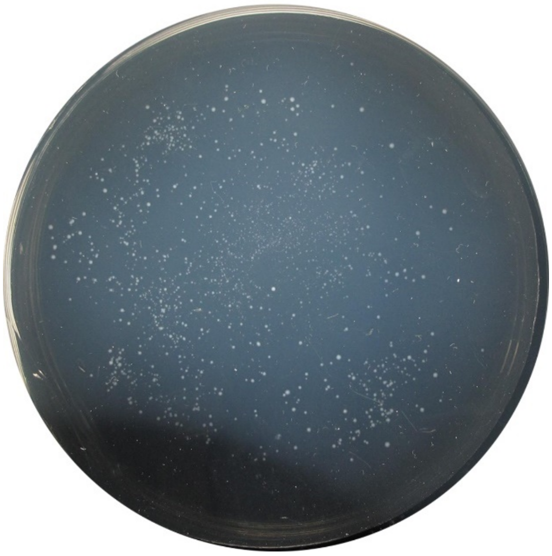

Figure S3. Base editing of the *upp^{6tgg}* variant (Related to Figure 1). The *upp^{6tgg}* edited clones can grow on 5-Fu plates after inducing BE.

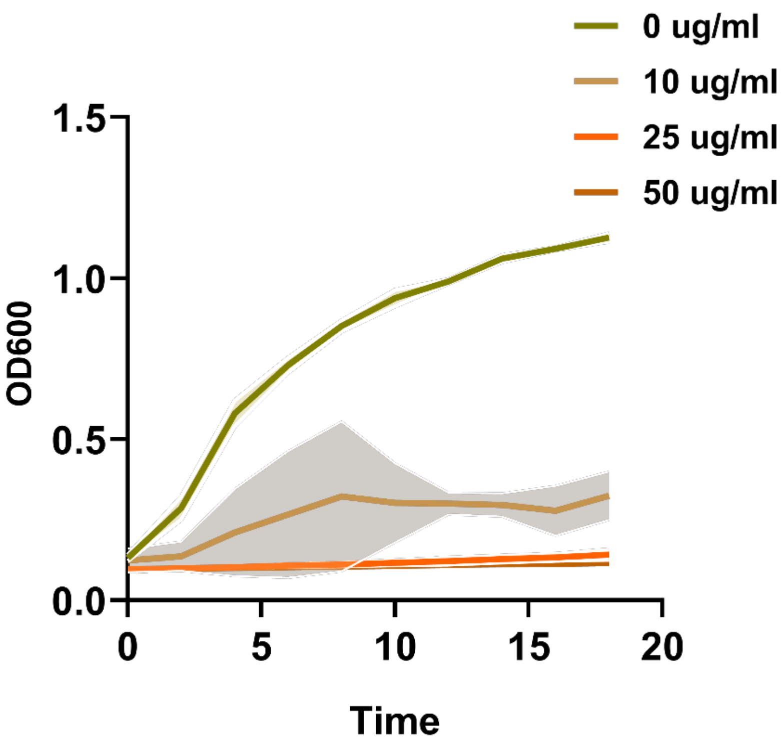

Figure S4. Growth curves of the strain GB05Red in various 5-Fu concentrations (Related to Figure 2).

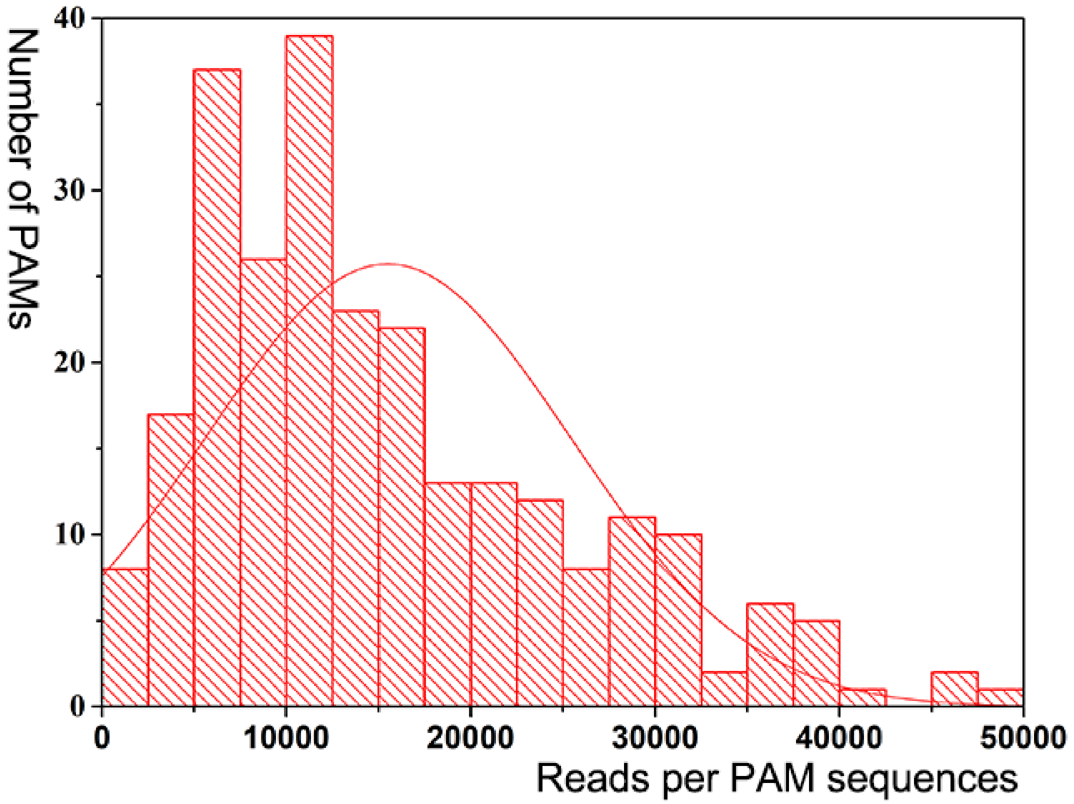

Figure S5. The diversity and abundance of PAM sequence in the initial PAM libraries (Related to Figure 3). NGS sequencing was performed for the initial PAM library, and the reads number of the 256 PAM sequences was statistically analyzed to ensure the quality of initial PAM library.

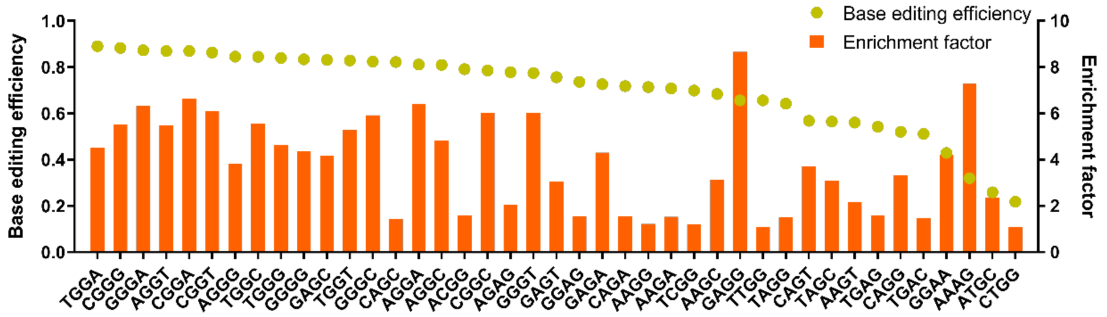

Figure S6. Functional PAM sequences with enrichment factor greater than 1 after two round of BESS screening (replicate 2) (Related to Figure 3). Brown points represent BE efficiencies and the histograms indicate enrichment factors.

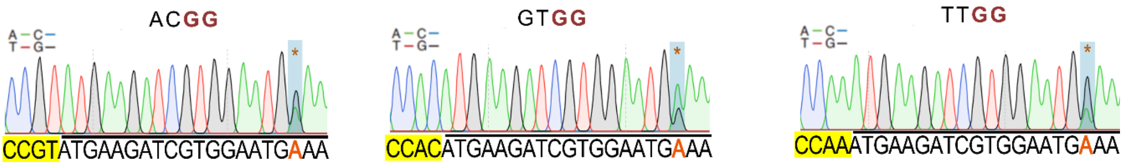

Figure S7. Sanger sequencing of *upp*^{6tgg} after BE using the ACGG, GTGG, and TTGG PAMs (Related to Figure 3).

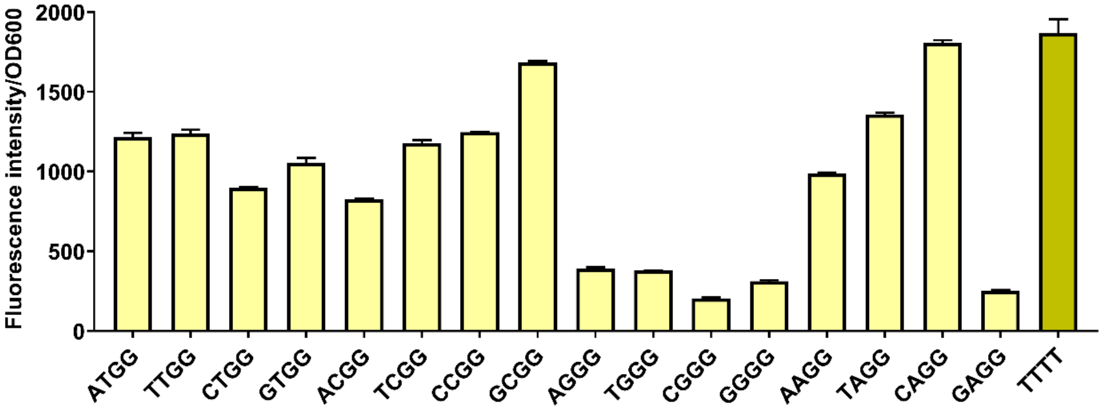

Figure S8. NNGG as PAM sequences to repress the expression of GFP by CRISPRi (Related to Figure 4).

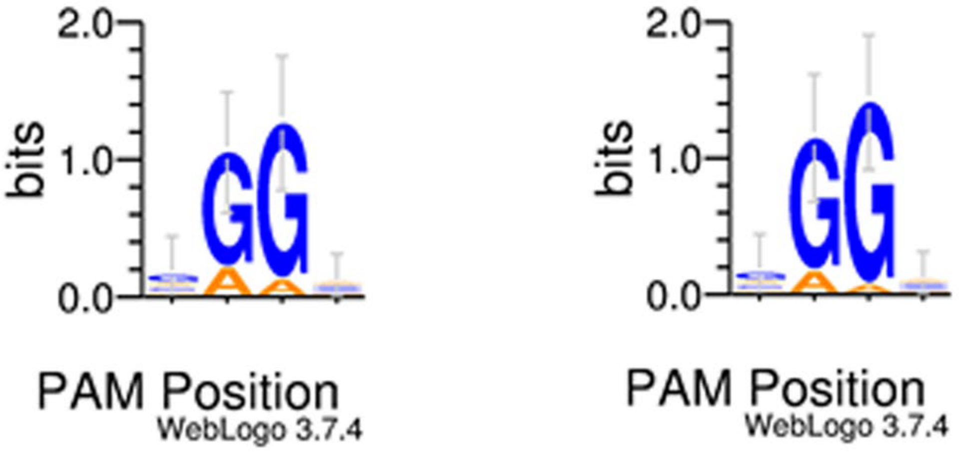

Figure S9. Sequence logos for the functional PAM sequences of SpdCas9 identified after two round of BESS screening (two replicates) (Related to Figure 5).

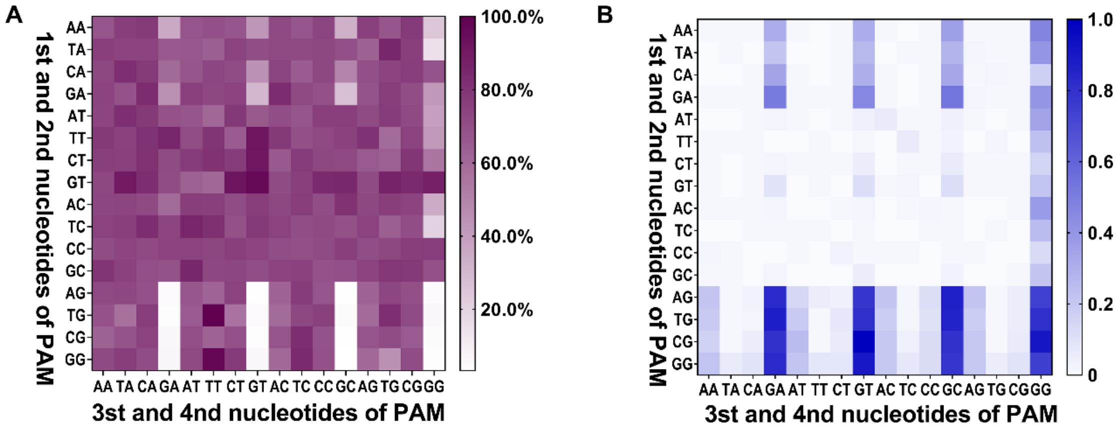

Figure S10. Heat map evaluation of the PAM compatibility with plasmid clearance experiment (A) and BE activity experiment (B) (Related to Figure 5).

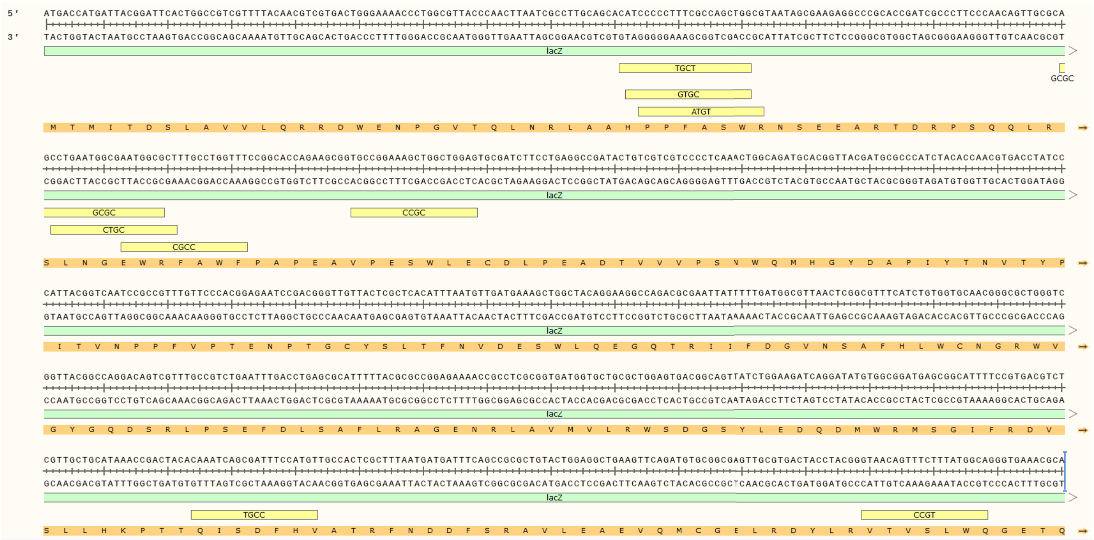

Figure S11. The distribution of sgRNA sequences with various PAMs on the *lacZ* gene (Related to Figure 5).

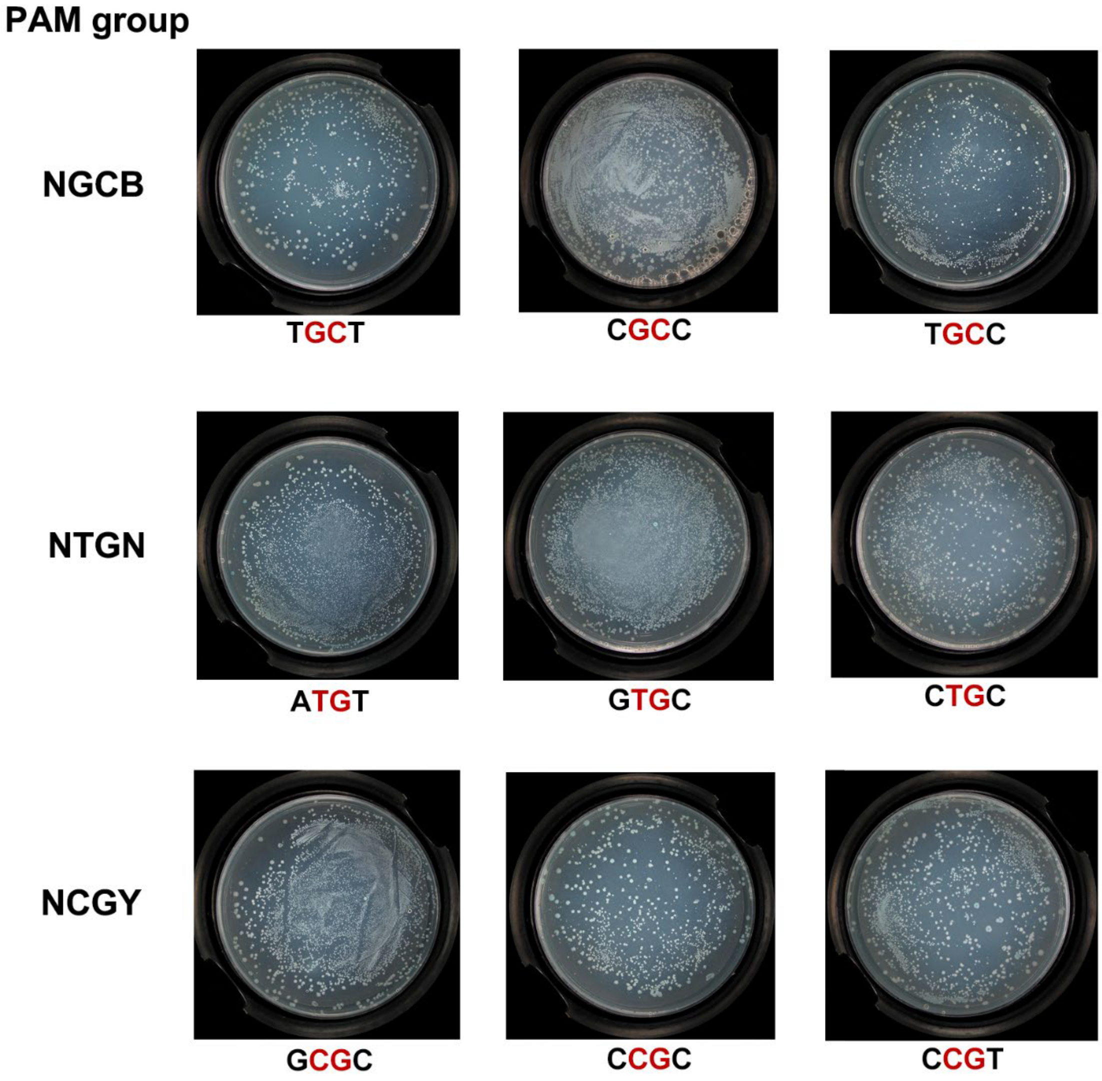

Figure S12. Blue-white screening of the *E. coli* MG1655 strains carrying sgRNA targeting *lacZ* gene with different PAMs after BE (Related to Figure 5).

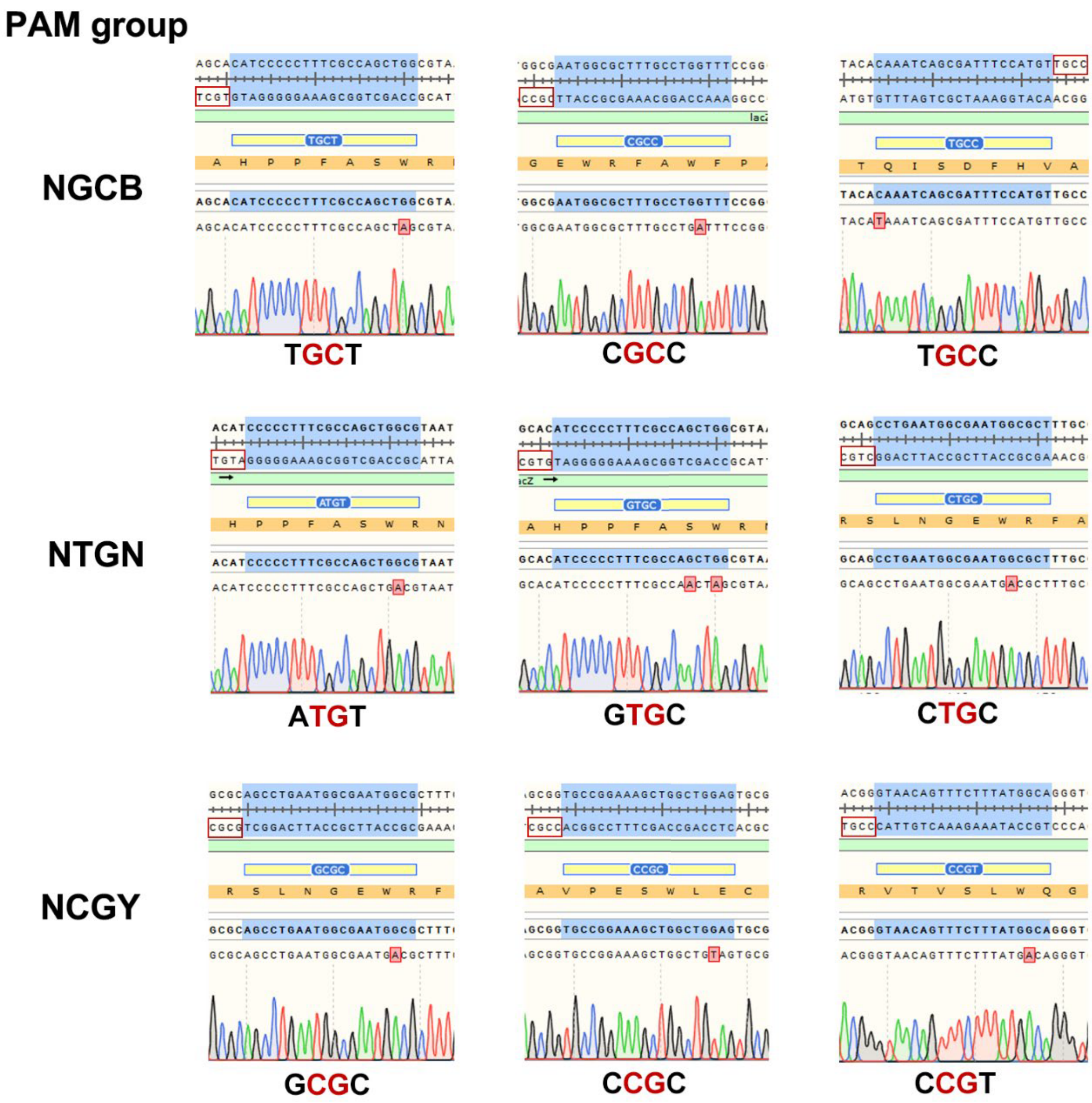

Figure S13. Sanger sequencing of the target sites on the genomic *lacZ* gene after BE using sgRNA with different PAMs (Related to Figure 5). The PAM sequence is noted with a red box in the figure.

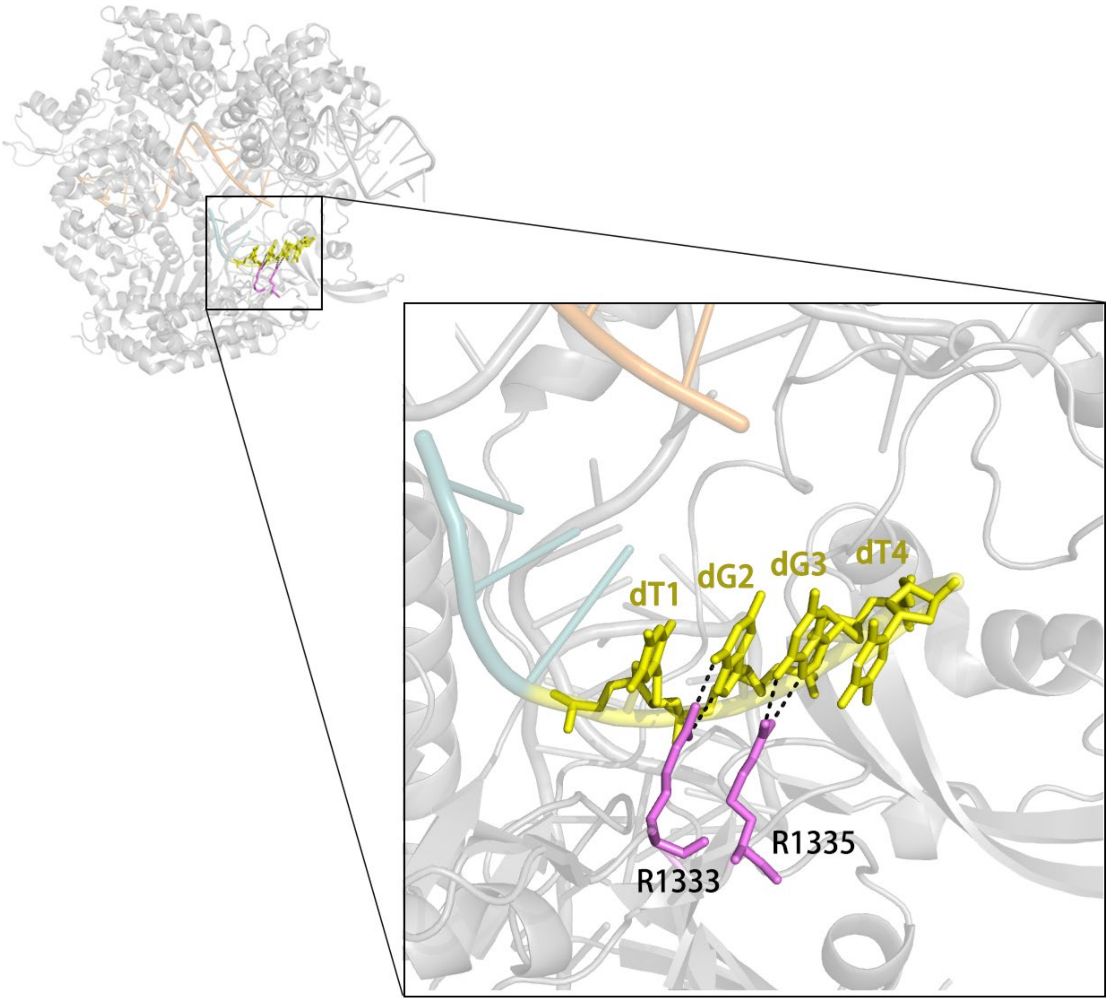

Figure S14. Interaction of arginine at the 1333 and 1335 positions in SpCas9 with NGG PAM (Related to Figure 5).
